# Prevalence, drug education, and other associated factors of current illicit drug use among a nationally representative sample of school-aged adolescents in the Philippines in 2019

**DOI:** 10.1186/s13011-025-00652-y

**Published:** 2025-05-22

**Authors:** Supa Pengpid, Karl Peltzer, Lyndon Esconde Santos, Earl Francis Infante Mallari

**Affiliations:** 1 Department of Health Education and Behavioral Sciences, Faculty of Public Health, Mahidol University, 420/1 Ratchawithi Road, Ratchathewi, Bangkok, 10400 Thailand; 2 Department of Public Health, Sefako Makgatho Health Sciences University, Pretoria, South Africa; 3 Department of Healthcare Administration, College of Medical and Health Science, Asia University, Taichung, Taiwan; 4 Department of Psychology, University of the Free State, Bloemfontein, South Africa; 5 Department of Psychology, College of Medical and Health Science, Asia University, Taichung, Taiwan; 6 Department of Healthcare Management Specialty in Psychology, Asia University, Taichung, Taiwan; 7 Department of Psychology, Far Eastern University, Manila, Philippines

**Keywords:** Drug use, Cannabis, Methamphetamine, Drug education, Adolescents, Philippines

## Abstract

**Background:**

The aim of the study was to assess the prevalence, drug education, and associated individual level, family and peer level, school level and community/macro level risk/protective factors of current illicit drug use among in-school adolescents in the Philippines.

**Method:**

The 2019 Philippines Global School-based Student Health Survey (GSHS), a nationally representative survey of teenagers aged 11 to 18 (mean age 13.8 years, Standard Deviation-SD = 1.5) that used a multistage sampling technique, provided the study’s data. Past 30-day illicit drug use, including cannabis, methamphetamine, ecstasy, rugby (a contact cement used as an adhesive which contains Toluene), and cocaine, was assessed by self-report. In order to determine the variables associated with current illicit drug use, the study used bivariate and multivariable logistic regression analysis.

**Results:**

The proportion of current illicit drug use was 14.1%, 8.6% among girls and 19.1% among boys. In the final adjusted model in relation to individual level risk/protective factors found that male sex (Adjusted Odds Ratio-AOR = 1.81, 95% Confidence Interval-CI = 1.45–2.28), food insecurity (AOR = 1.58, 95% CI = 1.33–1.88), psychological distress (AOR = 1.40, 95% CI = 1.10–1.77), current alcohol use (AOR = 2.14, 95% CI = 1.81–2.51) were positively associated and older age (15–18 + years) (AOR = 0.59, 95% CI = 0.45–0.77) was negatively associated with current drug use. In terms of family and level factors, high parental support (AOR = 0.45, 95% CI = 0.32–0.63), having close friends (AOR = 0.55, 95% CI = 0.38–0.80) and peer support (AOR = 0.65, 95% CI = 0.51–0.81) were all negatively associated with current drug use. Regarding school level factors, having been taught where to get help for drug problems (AOR = 0.77, 95% CI = 0.62–0.94) was inversely associated and having been taught about drug problems was marginally significantly negatively associated with current drug use. Furthermore, school truancy (AOR = 1.80, 95% CI = 1.43–2.27) was positively associated with current drug use. Community/macro level factors found that participation in physical fighting (AOR = 1.56, 95% CI = 1.24–1.97), and “someone offered, sold, or given you a drug,” (AOR = 5.40, 95% CI = 4.42–6.74) were positively associated with current drug use.

**Conclusion:**

One in seven Filipino adolescents engaged in current illicit drug use in 2019. Protective factors (such as high parental and peer support) and drug education were negatively associated with current illicit drug use. Individual and community level factors (such as psychological distress, exposure to drugs, alcohol use, and interpersonal violence) were positively associated with current illicit drug use. School and community programmes and policies may target to decrease psychosocial stressors, promote protective factors, and enhance curriculum-based drug education among adolescents in the Philippines.

## Introduction

Drug use among teenagers is a serious worldwide issue [1] and can be harmful at any level. Adolescent drug use is frequently linked to an increase in other risky behaviors in addition to the immediate health risks It can cause dependency to develop more quickly than it would in adulthood [2]. Cannabis use among people aged 15 to 16 as a yearly prevalence, was 5.6% in 2020 globally [2]. The prevalence of lifetime amphetamine use ranged from 1.2% in Laos to 4.8% in the Philippines and 5.1% in Timor-Leste, while the proportion of current use of cannabis was 3.1% in five Southeast Asian countries, ranging from 0.4% in Laos to 5.6% in the Philippines [3]. Among male adolescents in Malaysia, the proportion illicit drug use (lifetime) was 6.6% [4], and among 12-to-17-year-olds in 16 low- and middle-income countries (LMIC), the proportion of current cannabis use was 4.3% [5]. About 4.2% of school adolescents in the Philippines were co-users of cannabis and alcohol [6]. Among high school students (13–17 years) (*n* = 531) in Manila [7], 4.7% used illegal drugs (“methamphetamines, marijuana, and ecstasy”), 12.3% were provided or sold a drug within the previous 30 days, and 62.5% had ever been taught about “the dangers of using drugs.” According to the Republic Act (RA) no. 11,036 [8] also known as the Mental Health Law in the Philippines, schools are obliged to have programmes to raise awareness as well as to identify, manage or refer students with mental health problems [8]. Furthermore, according to the Philippines RA 9165 [9], schools are to have programmes for the prevention and deterrence of illicit drug use as well as conduct random drug testing. In addition, teachers are encouraged to report anyone that violates this legislation to legal authorities [9]. It is unclear, however, to what extent mental health promotion, including drug education, has been implemented in schools and if it had an impact on drug use, which led to this study.

Factors associated with adolescent drug use can be classified into the following categories using a socio-ecological model [10]: individual level risk/protective factors.

(age, sex, food insecurity, religiosity, psychological distress, and alcohol use), family and peer level risk/protective factors (parental support, having close friends, and peer support), school level risk/protective factors (type of school, exposure to drug education, and school truancy) and community/macro level risk/protective factors (bullying victimization, aggressive behaviour, and drug exposure) [3, 4, 11–13]. According to the socio-ecological model, environmental factors that affect behavior can be divided into four main categories: micro-systems, which include factors at the individual, family, and peer levels; meso-systems, which include factors at the school level; exo-systems; and macro-systems, which include factors at the community and macro level. Drug use behaviour is determined by interactions within and between these domains [10]. The study’s objective was to evaluate the prevalence, drug education, and related factors of current illicit drug use among Filipino school-age adolescents.

## Method

### Data source

This paper uses data from the Global School-based Student Health Survey (GSHS) conducted in the Philippines in 2019, which is the fifth iteration of the survey in the country. The GSHS is a self-administered, nationally representative survey that assesses a wide range of behaviours including protective factors, mental health, and alcohol use among other modules [14]. The sample was selected using a two-stage cluster sample design, and the 2019 survey had an overall response rate of 85% in the Philippines. All students in the selected classrooms were eligible to participate in the survey regardless of age. Student privacy was protected through anonymous and voluntary participation. The inquiry is filled out anonymously using a self-administered answer form. The completed computer-scannable response sheets are processed by the data coordinating centre [15]. The study was authorized by a national ethics council, and informed consent was obtained from participants or their guardians before the survey was administered.

### Study variables (see Table 1)

When the GSHS questionnaires were initially developed in English and Spanish, the participating countries translated and verified them into their own languages [15]. Core, core-expanded, and country-specific questions in the relevant language are used to construct country-specific questions for a self-administered questionnaire [15]. The GSHS is comparable to the “CDC Youth Risk Behavior Survey,” for which test- and retest reliability has been proven [16]. Furthermore, the GSHS questionnaire demonstrated a 77% test–retest consistency, according to Becker et al. [17].

#### Outcome variable

Current drug use was assessed with the question, “During the past 30 days, what was the last drug/substance that you used?” (Response options were “1. I did not use any drug/substance in the past 30 days, 2. Marijuana, 3. Shabu (methamphetamine), 4. Ecstasy, 5. Rugby and 6. Cocaine”).

**Individual level risk/protective factors** included age, sex, hunger, or food insecurity, whether the student is religious or spiritual, current alcohol use, and psychological distress. Psychological distress was assessed using five item indicators namely, suicide ideation, suicide plan, suicide attempt, anxiety, and loneliness; items were summed and dichotomized into 0–1 (= low) and 2–4 having high psychological distress. The 5-item psychological distress measure had a Cronbach alpha of 0.70 in this study.

### Family and peer level risk/protective factors

Parental support included four items, including parental supervision, connectedness, bonding, and parental respect; items were summed and grouped into 0–1 = low, 2 = moderate, and 3–4 = high parental support. Cronbach alpha for this parental support index was 0.66 in this sample. Peer support and having close friends were assessed each with one item.

**School level risk/protective factors** included type of school, school truancy, and exposure to drug education. Drug education included two items, including (1) “During this school year, were you taught in any of your classes the problems associated with using drugs such as marijuana, shabu, ecstasy, cocaine or rugby?” (Yes/No or do not know), and (2) “During this school year, were you taught in any of your classes where to get help to stop using drugs such as marijuana, shabu, ecstasy, cocaine, or rugby?” (Yes/No or do not know).

**Community/macro level risk/protective factors** included being bullied (past 12-month bullied on school property, when you were not on school property, or cyber bullied through messaging, Instagram, Facebook, or other social media), being physically attacked, getting in a physical fight, and someone offered, sold or gave you a drug.

The coding of the variables is also presented in Table 1.

**Table 1:** Description of global School-based student health survey (GSHS) study variables

Variables	Question	Response options (coding scheme)
Current drug use	“During the past 30 days, what was the last drug/substance that you used?”	1 “I did not use any drug/substance in the past 30 days” 2 “Marijuana” 3 “Shabu” 4 “Ecstasy” 5 “Rugby” 6 “Cocaine”
	**Individual level risk/protective factors**	
Age	“How old are you?”	“11 years old or younger to 16 or years old or older”
Sex	“What is your sex?”	“Male, Female”
Food insecurity	“During the past 30 days, how often did you go hungry because there was not enough food in your home?”	“1 = never to 5 = always” “(coded 1 = 0, 2–3 = 2 and 4–5 = 1)”
Is religious or spiritual	“Do you think of yourself as a religious or spiritual person?”	1 = Yes and 0 = No
“Loneliness”	“During the past 12 months, how often have you felt lonely?”	“1 = never to 5 = always (coded 1–3 = 0 and 4–5 = 1)”
“Worry-induced sleep disturbance”	“During the past 12 months, how often have you been so worried about something that you could not sleep at night?”	“1 = never to 5 = always (coded 1–3 = 0 and 4–5 = 1)”
“Suicidal ideation”	“During the past 12 months, did you ever seriously consider attempting suicide?”	“Yes, No”
“Suicide plan”	“During the past 12 months, did you make a plan about how you would attempt suicide?”	“Yes, No”
“Suicide attempt”	“During the past 12 months, how many times did you actually attempt suicide?”	“1 = 0 times to 5 = 6 or more times” (coded: 1 = 0 and 2–5 = 1)
“Current alcohol use”	“During the past 30 days, on how many days did you have at least one drink containing alcohol?”	“1 = 0 days to 7 = All 30 days” “(coded 1 = 0 and 2–7 = 1)”
	**Family and peer level risk/protective factors**	
“Parental supervision”	“During the past 30 days, how often did your parents or guardians check to see if your homework was done?”	“1 = never to 5 = always (coded 1–3 = 0 and 4–5 = 1)”
“Parental connectedness”	“During the past 30 days, how often did your parents or guardians understand your problems and worries?”	“1 = never to 5 = always (coded 1–3 = 0 and 4–5 = 1)”
“Parental bonding”	“During the past 30 days, how often did your parents or guardians really know what you were doing with your free time?”	“1 = never to 5 = always (coded 1–3 = 0 and 4–5 = 1)”
“Parental respect”	“During the past 30 days, how often did your parents or guardians go through without your approval?”	“1 = never to 5 = always (coded 3–5 = 0 and 1–2 = 1)”
“Close friends”	“How many close friends do you have?”	“1 = 0 to 4 = 3 or more (coded 1 + = 1, 0 = 0)”
“Peer support”	“During the past 30 days, how often were most of the students in your school kind and helpful?”	“1 = never to 5 = always (coded 1–3 = 0 and 4–5 = 1)”
	**School level risk/protective factors**	
Type of school	“Are you going to a public or private school now?”	1 = Public and 0 = Private
Taught about drug use problems	“During this school year, were you taught in any of your classes the problems associated with using drugs such as marijuana, shabu, ecstasy, cocaine or rugby?”	1 = Yes, 0 = No or do not know
Taught where to get help for drug problems	“During this school year, were you taught in any of your classes where to get help to stop using drugs such as marijuana, shabu, ecstasy, cocaine, or rugby?”	1 = Yes, 0 = No or do not know
“School truancy”	“During the past 30 days, on how many days did you miss classes or school without permission?”	“1 = 0 days to 5 = 10 or more days (coded 1 = 0 and 2–5 = 1)”
	**Community/macro level risk/protective factors**	
“Bullied”	“During the past 12 months, have you ever been bullied on school property?”	“Yes, No”
	“During the past 12 months, have you ever been bullied when you were not on school property?”	“Yes, No”
	“During the past 12 months, have you ever been cyber bullied (Count being bullied through messaging, Instagram, Facebook, or other social media.)?”	“Yes, No”
“Physically attacked”	“During the past 12 months, how many times were you physically attacked?”	“1 = 0 times to 8 = 12 or more times (coded 1 = 0 and 2–8 = 1)”
“In a physical fight”	“During the past 12 months, how many times were you in a physical fight?”	“1 = 0 times to 8 = 12 or more times (coded 1 = 0 and 2–8 = 1)”
“Someone offered, sold, or given you a drug”	“During the past 30 days, has anyone offered, sold, or given you a drug, such as marijuana, shabu, ecstasy, cocaine or rugby?”	“Yes, No”

### Data editing and analysis

All data processing, including scanning, cleaning, editing, and weighting, was carried out at the US Centers for Disease Control and WHO. Data processing comprises missing data management, including completeness checks, and plausibility checks for certain variables [15].

To describe the prevalence of the variables in the study, descriptive statistics was conducted using frequencies and percentages for all variables in the study. In order to estimate a nationally representative sample, cases were weighted using the recommended weighing factor. Collinearity was checked using Variance Inflation Factor (Minimum VIF = 1.05; Maximum VIF = 1.28). Binary logistic regression was further performed to test for the association between the predictor variables and the outcome variable. Independent variables were included based on literature review [3, 4, 10–13]. Only the significant predictors (*p* < 0.05) in the univariate analysis were included in the multivariable analysis. The complex sampling was taken into consideration when performing statistical analyses using STATA software version 18.0 (Stata Corporation, College Station, TX, USA).

## Results

### Sample characteristics

The total sample included 10,175 students (mean age = 13.8 years, Standard Deviation = 1.5). The proportion of current illicit drug use was 14.1%, 8.6% among girls and 19.1% among boys. In all, 44.4% of students had been taught about “the problems associated with using drugs such as marijuana, shabu, ecstasy, cocaine or rugby” in the current school year, 46.7% had been taught “where to get help to stop using drugs such as marijuana, shabu, ecstasy, cocaine, or rugby” in this school year, and 15.7% of the students had been “offered, sold, or given you a drug, such as marijuana, shabu, ecstasy, cocaine or rugby” by someone in the past 30 days (see Table 2).

**Table 2:** Sample characteristics and current drug use among high school adolescents in the Philippines, 2019

Variables	Classification	Sample	Current drug use
		*N* (%)	*N* (%)
All		10,175	1361 (14.1)
***Individual level risk/protective factors***			
Age	≤ 11–14	6653 (65.5)	1014 (16.3)
	15–18+	3496 (34.5)	337 (9.7)
Sex	Female	5421 (50.5)	444 (8.6)
	Male	4686 (49.5)	877 (19.1)
Food insecurity	No	8799 (87.3)	1053 (12.7)
	Yes	1356 (12.7)	298 (23.3)
Is religious or spiritual	No	3008 (33.0)	416 (14.4)
	Yes	6540 (67.0)	634 (10.3)
Psychological distress	0–1	7382 (72.2)	809 (11.4)
	2–5	2792 (27.8)	551 (21.1)
Current alcohol use	No	7518 (75.4)	624 (8.4)
	Yes	2319 (24.6)	575 (26.4)
***Family and peer level risk/protective factors***			
Parental support	0–1	5797 (57.2)	953 (17.2)
	2	2356 (23.8)	307 (13.6)
	3–4	1963 (19.0)	100 (5.6)
Close friends	No	660 (6.8)	196 (32.6)
	Yes	9404 (93.2)	1117 (12.4)
Peer help/ support	No	6647 (66.3)	1026 (16.1)
	Yes	3378 (33.7)	289 (9.2)
***School level risk/protective factors***			
Type of school	Private	2023 (19.3)	267 (14.4)
	Public	7843 (80.7)	1009 (13.5)
Taught about drug use problems	No	5525 (55.6)	773 (14.6)
	Yes	4377 (44.4)	483 (11.5)
Taught where to get help for drug problems	No	5238 (53.3)	765 (15.1)
	Yes	4626 (46.7)	482 (10.8)
School truancy	No	6910 (67.4)	560 (8.7)
	Yes	3144 (32.6)	757 (24.4)
**Community/macro level risk/protective factors**			
Bullied	No	5024 (51.3)	548 (11.3)
	Yes	5030 (48.7)	747 (15.9)
Physically attacked	No	6895 (68.0)	700 (10.7)
	Yes	3217 (32.0)	649 (21.4)
In physical fight	No	6665 (65.8)	577 (8.9)
	Yes	3420 (34.2)	759 (23.9)
Someone offered, sold, or given you a drug	No	8702 (84.3)	540 (7.1)
	Yes	1454 (15.7)	473 (33.1)

### Prevalence of current drug use types

Overall, the most commonly drug used in the past 30 days was cannabis (7.9%), followed by Shabu (methamphetamine) (3.6%), ecstasy (1.3%), rugby (a contact cement used as an adhesive which contains Toluene) [18] (0.8%) and cocaine (0.5%). The use of all drugs was higher in male and younger adolescents (see Table 3).

**Table 3:** Type of current drug use among high school adolescents in the Philippines, 2019

Variable	Type of drug/substance use in the past 30 days						*p*-value
	None	Cannabis (Marijuana)	Shabu	Ecstasy	Rugby	Cocaine	
	N (%)	N (%)	N (%)	N (%)	N (%)	N (%)	
All	8664 (85.9)	762 (7.9)	337 (3.6)	131 (1.3)	79 (0.8)	52 (0.5)	
Female	4898 (91.4)	262 (5.0)	92 (1.9)	46 (0.8)	22 (0.4)	22 (0.4)	< 0.001
Male	3738 (89.9)	482 (10.6)	230 (5.0)	81 (1.7)	55 (1.2)	29 (0.6)	
≤ 11–14	5546 (83.7)	559 (8.9)	251 (4.2)	102 (1.6)	59 (1.0)	43 (0.7)	< 0.001
15–18+	3102 (90.3)	196 (5.9)	84 (2.3)	28 (0.7)	20 (0.5)	9 (0.3)	

### Associations with current drug use

#### Individual level risk/protective factors

In the final adjusted model in relation to individual level risk/protective factors found that male sex (Adjusted Odds Ratio-AOR = 1.81, 95% Confidence Interval-CI = 1.45–2.28), food insecurity (AOR = 1.58, 95% CI = 1.33–1.88), psychological distress (AOR = 1.40, 95% CI = 1.10–1.77),

current alcohol use (AOR = 2.14, 95% CI = 1.81–2.51) were positively associated and older age (15–18 + years) (AOR = 0.59, 95% CI = 0.45–0.77) was negatively associated with current drug use.

#### Family and peer level risk/protective factors

High parental support (AOR = 0.45, 95% CI = 0.32–0.63), having close friends (AOR = 0.55, 95% CI = 0.38–0.80) and peer support (AOR = 0.65, 95% CI = 0.51–0.81) were all negatively associated with current drug use.

#### School level risk/protective factors

Regarding drug education, having been taught where to get help for drug problems (AOR = 0.77, 95% CI = 0.62–0.94) was inversely associated and having been taught about drug problems was marginally significantly negatively associated with current drug use. Furthermore, school truancy (AOR = 1.80, 95% CI = 1.43–2.27) was positively associated with current drug use.

#### Community/macro level risk/protective factors

Participation in physical fighting (AOR = 1.56, 95% CI = 1.24–1.97), and “someone offered, sold, or given you a drug,” (AOR = 5.40, 95% CI = 4.42–6.74) were positively associated with current drug use (see Table 4).

**Table 4:** Univariable and multivariable logistic regression analysis on the predictors of current drug use among high school adolescents in the Philippines

Variables		cOR [95% CI]	*p*-value	aOR [95% CI]	
***Individual level risk/protective factors***					
Age	≤ 11–14	1 (Reference)		1 (Reference)	
	15–18+	0.55 (0.44 to 0.69)	< 0.001	0.59 (0.45 to 0.77)	< 0.001
Sex	Female	1 (Reference)		1 (Reference)	
	Male	2.51 (2.13 to 2.95)	< 0.001	1.81 (1.45 to 2.28)	< 0.001
Food insecurity	No	1 (Reference)		1 (Reference)	
	Yes	2.08 (1.78 to 2.43)	< 0.001	1.58 (1.33 to 1.88)	< 0.001
Is religious or spiritual	No	1 (Reference)		1 (Reference)	
	Yes	0.68 (0.59 to 0.79)	< 0.001	0.88 (0.73 to 1.07)	0.207
Psychological distress	0–1	1 (Reference)		1 (Reference)	
	2–5	2.07 (1.74 to 2.46)	< 0.001	1.40 (1.10 to 1.77)	0.007
Current alcohol use	No	1 (Reference)		1 (Reference)	
	Yes	3.90 (3.45 to 4.40)	< 0.001	2.14 (1.81 to 2.51)	< 0.001
***Family and peer level risk/protective factors***					
Parental support	0–1	1 (Reference)		1 (Reference)	
	2	0.76 (0.63 to 0.91)	0.004	0.81 (0.62 to 1.07)	0.133
	3–4	0.28 (0.21 to 0.37)	< 0.001	0.45 (0.32 to 0.63)	< 0.001
Close friends	No	1 (Reference)		1 (Reference)	
	Yes	0.29 (0.24 to 0.36)	**<** 0.001	0.55 (0.38 to 0.80)	0.002
Peer help/ support	No	1 (Reference)		1 (Reference)	
	Yes	0.53 (0.44 to 0.63)	< 0.001	0.65 (0.51 to 0.81)	< 0.001
***School level risk/protective factors***					
Type of school	Private	1 (Reference)			
	Public	0.93 (0.60 to 1.45)	0.742	---	
Taught about drug use problems	No	1 (Reference)		1 (Reference)	
	Yes	0.76 (0.64 to 0.91)	0.004	0.78 (0.60 to 1.00)	0.052
Taught where to get help for drug problems	No	1 (Reference)		1 (Reference)	
	Yes	0.68 (0.58 to 0.79)	< 0.001	0.77 (0.62 to 0.94)	0.011
School truancy	No	1 (Reference)		1 (Reference)	
	Yes	3.40 (2.95 to 3.93)	< 0.001	1.80 (1.43 to 2.27)	< 0.001
***Community/macro level risk/protective factors***					
Bullied	No	1 (Reference)		1 (Reference)	
	Yes	1.48 (1.26 to 1.75)	< 0.001	1.04 (0.84 to 1.27)	0.727
Physically attacked	No	1 (Reference)		1 (Reference)	
	Yes	2.29 (1.92 to 2.72)	< 0.001	1.24 (0.95 to 1.63)	0.107
In physical fight	No	1 (Reference)		1 (Reference)	
	Yes	3.22 (2.71 to 3.82)	< 0.001	1.56 (1.24 to 1.97)	< 0.001
Someone offered, sold, or given you a drug	No	1 (Reference)		1 (Reference)	
	Yes	6.51 (5.37 to 7.89)	< 0.001	5.40 (4.42 to 6.74)	< 0.001

cOR, crude Odds Ratio; aOR, adjusted Odds Ratio

## Discussion

The aim of the study was to assess the prevalence, drug education and associated factors of current illicit drug use in a nationally representative sample of school adolescents in the Philippines in 2019. We found that the estimated prevalence of illicit current drug use (14.1%) was higher than in previous studies in the Philippines (4.7–5.6% [3, 7], and the region, e.g., Malaysia (6.6%, lifetime illicit drug use among male adolescents [4]) and Timor-Leste (5.1%, lifetime amphetamine use [3], and in 16 LMIC (4.3% current cannabis use [5]). The increase of illicit current drug use (14.1%) among adolescents in this study in 2019 in the Philippines may be explained by the fact that the 2015 Philippines GSHS reports only current cannabis use (5.6%) [3], while in this study a more comprehensive measure of different illicit drugs was used; the prevalence of current cannabis use was in this study 7.9% which shows an increase by 2.3% of current cannabis use from 2015 to 2019. The only other study that assessed illegal drugs among high school students in a local study in Manila in 2008 [7] reported 4.7% use of illegal drugs, but only included “methamphetamines, marijuana, and ecstasy”, while our study in addition assessed rugby, and cocaine. Possible reasons for an increase in illicit drug use among adolescents in the Philippines can be explained by the fact that it is situated as a transit center for the illicit drug trade and a consumer market for illegal drugs [19], and that it is intertwined with “poverty, inequalities, poor access to health care, and systemic problems in governance.” [20]. For example, limited job opportunities drive those in poverty to the drug trade as a source of income. The widespread availability of these substances further exacerbates the problem, making drug use more common in struggling communities [21]. The increase in illicit drug use highlights the ineffectiveness of the “war on drugs” policy, which fails to recognize substance use as a public health issue and hinders access to essential health services for recovery [22]. Additionally, the lack of voluntary treatment options, overcrowding in compulsory rehabilitation centers, and reliance on spiritual and religious interventions have proven ineffective in reducing drug use in the country [23]. Moreover, young people employ everyday tactics (“*diskarte*”) to evade law enforcement and continue using drugs, which may further explain why drug use rates fail to decrease in the Philippines, especially among the youth [24]. Compared to a previous study among school adolescents in Manila, Philippines, that reported 12.3% were “given or sold a drug in the past 30 days”, and 62.5% had ever been “taught about the dangers of drugs” [7], in this study, 15.7% of the students had been “offered, sold, or given you a drug, such as marijuana, shabu, ecstasy, cocaine or rugby” by someone in the past 30 days, 44.4% of students had been taught about “the problems associated with using drugs such as marijuana, shabu, ecstasy, cocaine or rugby” in the current school year, and 46.7% had been taught “where to get help to stop using drugs such as marijuana, shabu, ecstasy, cocaine, or rugby”.

Having been exposed to drug education, especially “where to get help using drugs” was negatively associated with current illicit drug use. It appears that the integration of drug use prevention and control in the school curriculum, and possibly random drug testing among secondary school students [8] has been beneficial, and should be expanded to reach all school children. There is limited evidence that universal and targeted school-level interventions are beneficial for preventing illicit drug use among adolescents [25]. According to the Dangerous Drugs Board (DDB) in the Philippines [26], a wide range of awareness and control programmes on drug use are implemented. In 2021, the Philippines Department of Education launched its Preventive Drug Education Program (PDEP) Curriculum Model, aiming to provide classroom-based comprehensive drug use prevention efforts from Kindergarten to Grade 12 [27].

In line with earlier research [28], we discovered that boys were more likely than girls to use drugs currently. The gender gap may be explained by social norms that stigmatize girls more than boys who use cannabis and by the fact that boys tend to take greater risks than girls [28, 29].

Drug use did not increase with age in five Asian countries (Iraq, Kuwait, Malaysia, Mongolia, and Vietnam) [29], and Pacific nations (Cook Islands, Kiribati, Samoa, Solomon Islands, Tonga and Tuvalu) [28], but current cannabis use increased with age in eight African countries [30], while this study showed a significantly lower rate of illicit drug use among older adolescents (9.7%) than younger adolescents (16.3%). Possible reasons for this include that in this study (analysis not shown) the exposure to drug education was significantly lower in younger adolescents (40.2%) than older adolescents (52.4%), and the provision or sale of drugs was significantly higher in younger adolescents (17.1%) than older adolescents (13.0%). Of concern is the apparently ease of accessibility and availability of illicit drugs, especially in younger adolescents, as found in some other countries [12]. Among adolescents in five ASEAN countries (Indonesia, Laos, Philippines, Thailand, and Timor-Leste), adolescents in the Philippines had the highest prevalence of early onset (< 12 years) drug use (7.4%) [31]. Consequently, drug education prevention efforts may be intensified targeting younger adolescents (11–14 years).

Furthermore, the study found protective factors [parental and peer support and having close friends) against illicit drug use, which is consistent with previous studies [3, 32, 33]. Adolescents may not develop a habit of using illegal drugs if parents watch over them and show them support and concern through bonding and monitoring behaviours [34], and have social support by their peers and close friends. Strategies should be encouraged that can provide support in parenting programmes, in promoting favourable peer associations and social skills training, as part of the implementation of the Mental Health Act in schools, since drug dependence is explicitly stated as a mental health problem. Mental health promotion in schools may include educating people about mental health concerns, identifying and helping those who are at risk, and providing facility access, including ways to refer people with mental health conditions to treatment and psychosocial support [8].

Alcohol use was highly associated with current drug use among in-school adolescents in this study, demonstrating the co-use of alcohol and illicit drug use may have potential additive effects [35, 36].

Consistent with previous research [12, 29, 30, 33], psychological distress was associated with current illicit drug use in this study. Adolescents experiencing psychological distress may choose to use illicit drugs as a coping mechanism [3, 22], or they may use cannabis and/or amphetamine to change their state of mind [37].

In line with former studies [29, 30] other social stressors (physical fighting and school truancy) were associated with illicit drug use in this study. Reduced school connectivity among adolescents enrolled in school may account for the positive correlation between drug use and school truancy, thereby raising the risk of substance use [3].

### Study limitations

Since the study was cross-sectional, conclusions about causality cannot be drawn. Furthermore, the results may not be entirely representative of all Filipino adolescents, especially those who are not enrolled in school and may exhibit distinct drug use patterns, as a result of the exclusion of adolescents who are not enrolled in school. The measure of current illicit drug use (past-30-day drug use) only measured a binary yes/no for each substance, hindering us to measure to what extent adolescents are using the specific substances. Drug use was evaluated based on self-report, which may have introduced biased responses or recall bias, in particular considering the illegality of dangerous drug use, such as methamphetamine hydrochloride or “shabu”, cannabis and ecstasy, in the Philippines [38]. Possible strategies to mitigate these biases in future research to strengthen the study’s validity may include increasing the anonymity in the survey method, such as conducting the survey online without revealing personal identifiers. A few study variables, such as tobacco use, parental substance use, and sexual risk behaviour, were not assessed in the 2019 Philippines GSHS, and could therefore not included in the analysis. A number of study indicators were evaluated using single items; more thorough measures should be used in subsequent research.

## Conclusion

The study showed that one in seven school-going adolescents in the Philippines engaged in current illicit drug use (cannabis, shabu/amphetamine, ecstasy, rugby, or cocaine) in 2019. Protective factors (having close friends, peer, and parental support) and drug education (taught where to get help for drug problems, and taught about drug use problems) and school attendance were negatively associated and individual and community level factors (psychological distress, provision or sale of illicit drugs, current alcohol use, and involvement in physical fighting) were positively associated with current illicit drug use. School and community programmes and policies may target to decrease psychosocial stressors, promote protective factors, and enhance curriculum-based drug education among adolescents in the Philippines. To determine the causal relationships between drug education, protective and psychosocial factors with illicit drug use, more prospective studies are required.

## Data Availability

The data on which is paper is based is available at the World Health Organization, NCD Repository at https://extranet.who.int/ncdsmicrodata/index.php/catalog/944/get-microdata.

## Declarations

All methods were carried out in accordance with relevant guidelines and regulations and have been performed in accordance with the Declaration of Helsinki.

## Abbreviations

PDEP: Preventive Drug Education Program
GSHS: Global School-based Student Health Survey
LMIC: low- and middle-income countries
RA: Republic Act; Rugby (a contact cement used as an adhesive which contains Toluene)
Shabu: Methamphetamine
